# Plantar Vein Thrombosis Mimicking Tendinopathy

**DOI:** 10.5334/jbsr.2708

**Published:** 2022-01-25

**Authors:** Michaela Kubincova, Filip Vanhoenacker

**Affiliations:** 1 AZ Sint-Maarten, Mechelen, BE; 2 AZ Sint-Maarten and University (Hospital) Antwerp/Ghent, BE

**Keywords:** ankle, plantar vein, MRI, color Doppler ultrasound, venous thrombosis

## Abstract

**Teaching point**: Plantar vein thrombosis is a rare condition that should be considered in the differential diagnosis of ankle pain, particularly in patients with a high clinical index of suspicion for venous thrombosis.

## Case

**Figure 1:**
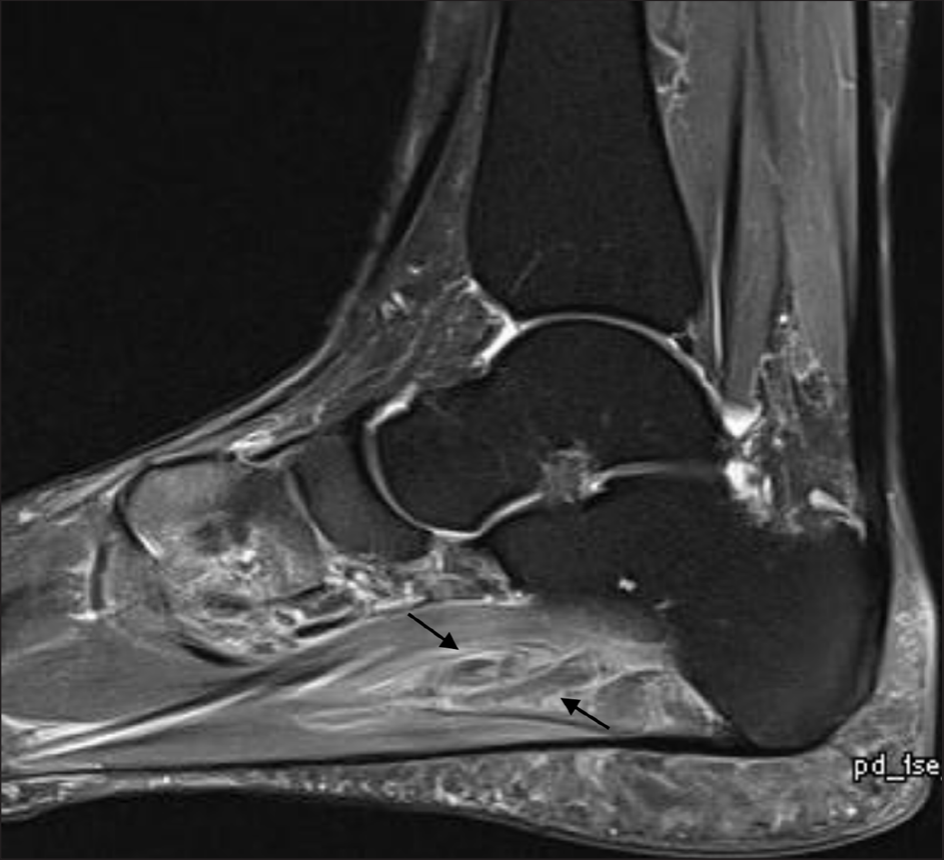


**Figure 2:**
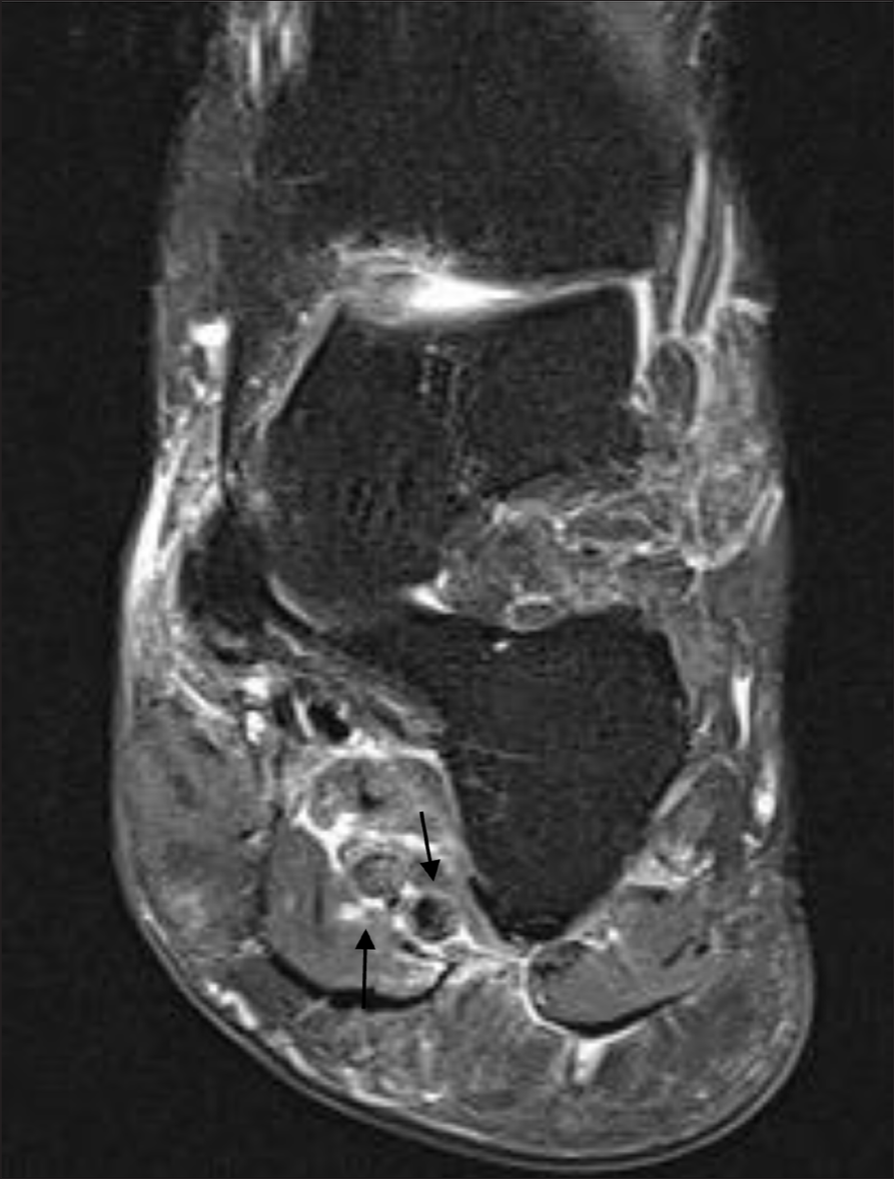


A 56-year-old female presented with left ankle pain and swelling that was aggravated while standing. The referring clinician suspected tibialis posterior tendinopathy. Magnetic resonance imaging (MRI) of the left ankle demonstrated absence of the normal high-intensity signal in the plantar veins on the sagittal (***Figure 1***, black arrows) and coronal (***Figure 2***, black arrows) fat-suppressed T2-weighted images (WI). There was muscle edema in quadratus plantae and flexor digitorum brevis muscle.

**Figure 3:**
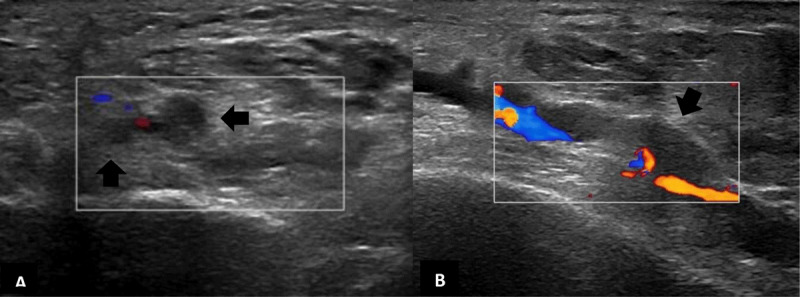


Subsequent color Doppler ultrasound examination showed absence of venous flow signal with intravenous hypo-echogenic clot formation within dilated noncompressible plantar veins (***Figure 3***, A coronal B sagittal, black arrows).

Repeated anamnesis revealed a known history of previous deep vein thrombosis, hereditary hypercoagulability disorder factor V Leiden, and usage of hormonal therapy and oral contraceptives.

## Comment

Plantar vein thrombosis is a rare condition that mostly affects the lateral plantar veins. Clinically, the disease presents with local swelling and pain increasing while walking or standing and plantar heel pain and may mimic other more common disorders of the ankle and foot such as tendinopathy or plantar fasciitis.

The risk factors include vessel wall damage, intravenous stasis of the blood, inappropriate footwear and hypercoagulable states caused by medication, neoplasia, postoperative states, trauma, and genetic disorders [1].

The deep plantar venous arch is created by the confluence of the metatarsal veins draining into the two deep medial and lateral plantar veins each accompanied by an artery and is connected by the perforating veins with the superficial dorsal venous arch. The confluence of the plantar veins is located at the level of the medial malleolus where the veins drain into the tibialis posterior veins.

The primary imaging modality to diagnose the deep plantar vein thrombosis is color Doppler ultrasonography, while MRI can be very useful in the obese patients and patient with a horny skin [1]. Due to the more frequent use of MRI nowadays, the disease is often detected first on MRI. It is important that the radiologist meticulously screens the plantar veins systematically on every MR examination of the ankle joint.

The treatment of choice consists of rest, elastic stockings, NSAIDs, and anticoagulants, which in the present case resulted in full recovery.

## References

[1] Vansevenant M, Vanhoenacker FM. Plantar vein thrombosis: An unusual cause of plantar pain. JBSR. 2015; 99(2): 98–101. DOI: 10.5334/jbr-btr.874 30039118 PMC6032566

